# Methods for Enhancing Clustered Regularly Interspaced Short Palindromic Repeats/Cas9-Mediated Homology-Directed Repair Efficiency

**DOI:** 10.3389/fgene.2019.00551

**Published:** 2019-06-17

**Authors:** Xi-Dian Tang, Fei Gao, Ming-Jie Liu, Qin-Lei Fan, De-Kun Chen, Wen-Tao Ma

**Affiliations:** ^1^Veterinary Immunology Laboratory, Department of Preventive Veterinary Medicine, College of Veterinary Medicine, Northwest Agriculture and Forestry University, Yangling, China; ^2^China Animal Health and Epidemiology Center, Qingdao, China

**Keywords:** genome editing, clustered regularly interspaced short palindromic repeats, homologous-directed repair efficiency, double-strand break, nonhomologous end joining

## Abstract

The evolution of organisms has provided a variety of mechanisms to maintain the integrity of its genome, but as damage occurs, DNA damage repair pathways are necessary to resolve errors. Among them, the DNA double-strand break repair pathway is highly conserved in eukaryotes, including mammals. Nonhomologous DNA end joining and homologous directed repair are two major DNA repair pathways that are synergistic or antagonistic. Clustered regularly interspaced short palindromic repeats genome editing techniques based on the nonhomologous DNA end joining repair pathway have been used to generate highly efficient insertions or deletions of variable-sized genes but are error-prone and inaccurate. By combining the homology-directed repair pathway with clustered regularly interspaced short palindromic repeats cleavage, more precise genome editing *via* insertion or deletion of the desired fragment can be performed. However, homologous directed repair is not efficient and needs further improvement. Here, we describe several ways to improve the efficiency of homologous directed repair by regulating the cell cycle, expressing key proteins involved in homologous recombination and selecting appropriate donor DNA.

## Introduction

### Clustered Regularly Interspaced Short Palindromic Repeats/CRISPR-Associated (Cas) Systems

Precise and efficient genomic modification is essential to biological processes, genetic engineering, and other various areas of study. In recent years, many techniques for mediating targeted genome editing have emerged throughout the world. The most important tools for genome editing are enzymes, including zinc finger nucleases, transcription activator-like effector nucleases, and engineered meganucleases (Richardson et al., 2016; Khadempar et al., 2018). A true revolution in genome editing occurred with the introduction of a programmable nuclease *via* the CRISPR-Cas system; Cas9 is one of the nucleases that plays a critical role during this process (Czarnek and Bereta, 2016). CRISPR-Cas technology can cleave specific DNA sequences (Jinek et al., 2012); endonucleases cleave DNA fragments precisely and efficiently through fusion with transcriptional activators and inhibitors by targeting histone-modifying enzymes for epigenetic regulation as well as manipulation of chromatin topologies for gene regulation (Adli, 2018).

Ishino et al. first identified CRISPR in *Escherichia coli* in 1987. They considered CRISPR as a gene editor, a system used by bacteria to protect themselves against viruses (Czarnek and Bereta, 2016). Later, researchers found that it appeared to be a precise genetic tool that could be used to delete, add, activate, or inhibit target genes in other organisms including humans, mice, bacteria, and fruit flies (Cong et al., 2013). The CRISPR cluster is a family of specific DNA repeats that are widely found in the genomes of bacteria and archaea, consisting of a leader, multiple short, and highly conserved repeat regions and multiple spacers (Dahlman et al., 2015). The leader region is generally located upstream of the CRISPR cluster and is an AT-rich region with a length of 300–500 bp; this is considered to be a promoter sequence of the CRISPR cluster. The repeat sequence region has a length of 21–48 bp and contains a palindromic sequence which can form a hairpin structure. In addition, the repeat sequences are separated by a spacer of 26–72 bp that consists of captured extraneous DNA, which is related to immune memory. When DNA containing the foreign sequence is encountered, it can be recognized by the bacteria and cut to inactivate the sequence in order to protect itself (Czarnek and Bereta, 2016). By analyzing the flanking sequence of the CRISPR cluster, it was found that there is a polymorphic family gene in its vicinity. The proteins encoded by this family contain functional domains (having nuclease, helicase, integrase, and polymerase activities) that interact with nucleic acids and work together with the CRISPR region; they are named CRISPR-associated (Cas) genes. Cas genes have been discovered, including Cas9. The Cas gene and CRISPR cluster have evolved together to form a highly conserved system, known as the CRISPR-Cas system (Salsman et al., 2017).

Subsequent studies showed that CRISPR and Cas9 endonuclease forms a complex, the gene encoding the Cas9 protein is located near the CRISPR locus, and that Cas9 creates a gap in the target DNA or RNA sequences. In addition, their genomes are protected from attack from phage nucleic acids and integrating plasmids by the CRISPR-Cas9 systems. In fact, CRISPR-Cas9 coordinates with the immune system and targets a wide range of invading proteins and nucleic acids such as RNA and DNA (Hale et al., 2009). Cas nucleases break down the invasive foreign DNA, part of which is placed in the CRISPR site between two repeated sequences (referred to as a spacer). The sequences of the spacer are further used as templates to produce short CRISPR RNAs (crRNAs; Jinek et al., 2012). These two sequences appear to act as a guide sequence to promote the binding of the Cas9 protein to the foreign DNA. Upon their successful binding, Cas9 protein cleaves invading DNA strands complementary to the crRNA sequence and its opposite sequence through the nuclease domains of HNH and RuvC, respectively (Jiang et al., 2016).

**Figure 1:**
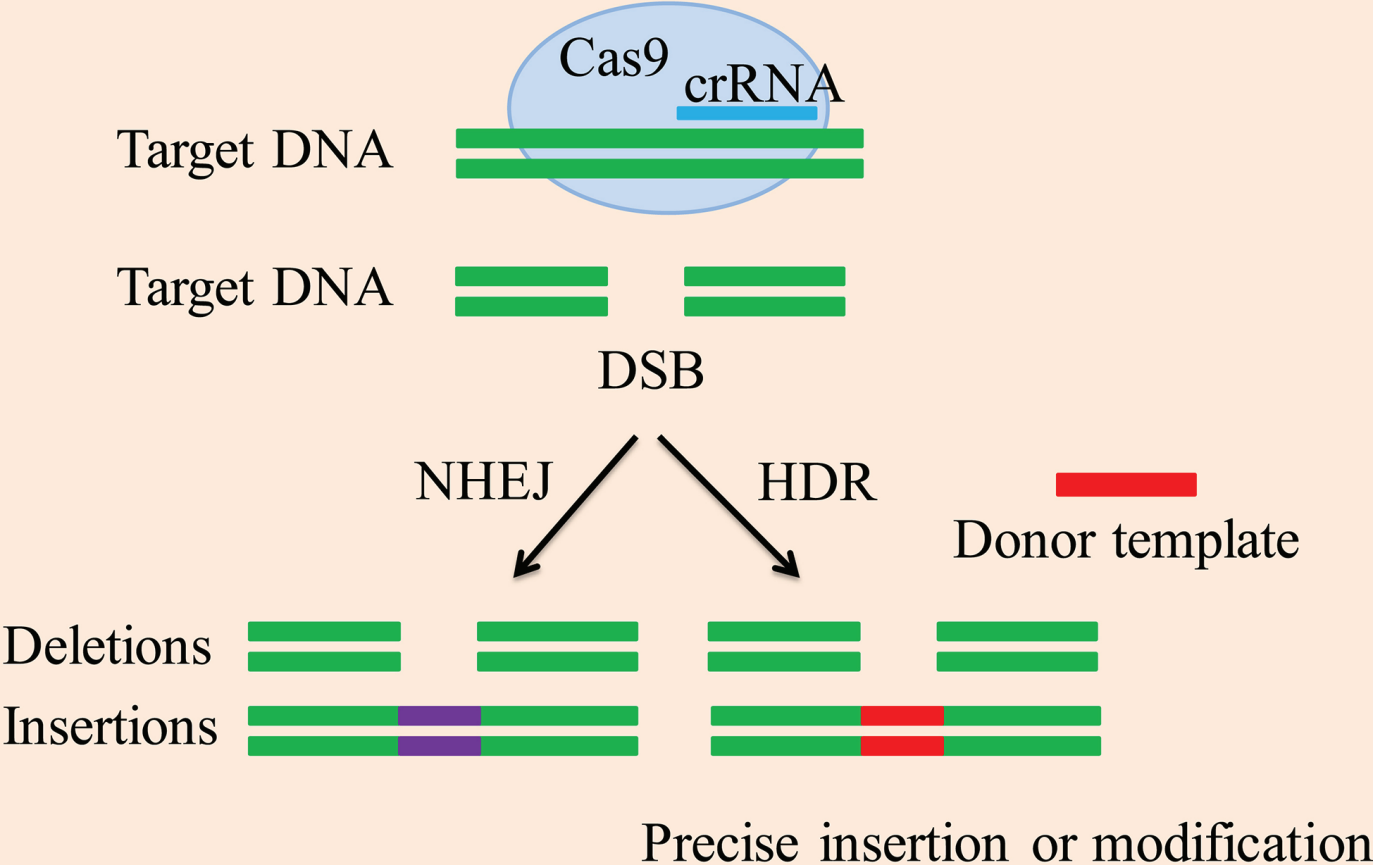
CRISPR/Cas9-mediated DSB repair mechanism. The CRISPR-associated enzyme Cas9 breaks down the target DNA to create a DSB, the two repeated sequences are further used as templates to produce short crRNAs. Methods for DSB repair include the NHEJ and HDR pathway. The NHEJ pathway creates accurate deletions and insertions. The HDR pathway uses homologous donor DNA sequences from sister chromatids or foreign DNA to create accurate insertions, base substitutions between DSB sites or two DSBs, and other modifications.

This system of genome editing can be used to select certain genetic products that have therapeutic potential. However, the editing of the specific sequences depends on the type of repair strategy being used by a cell, such as nonhomologous end joining (NHEJ) or homologous directed repair (HDR), as presented below in detail and summarized in Figure 1. The advantage of CRISPR technology is that it is very accurate, but the Cas9 protein sometimes removes sequences that are similar (not including the target sequence and off-target sequences). More precise control is required and is an area of further study (Hussain et al., 2018).

### Nonhomologous End Joining

The CRISPR-associated enzyme Cas9 achieves site-specific genomic engineering by introducing a double-strand break (DSB) at the chromosomal site specified by the guide RNA (Cong et al., 2013; Jinek et al., 2013; Mali et al., 2013). Cells repair the DSB using the NHEJ or HDR pathway. NHEJ is a major form of mammalian DNA repair machinery that successfully joins broken DNA together (Mao et al., 2008). The low fidelity of NHEJ, which is prone to errors, may result in a base deletion or insertion (indel) after repair, resulting in a frameshift mutation (Bernheim et al., 2017). Ultimately, the goal of gene knockout is achieved. Gene knockout model animals can be prepared by using a targeted nuclease to efficiently cause frameshift mutations at the fertilized egg level. The emergence of CRISPR/Cas9 technology makes it possible to prepare gene knockout model organisms without using the embryonic stem cell (ESC) line of the corresponding species and has been successfully applied to mice, rats, fruit flies, and the like (Gonzalez, 2016; Arnoult et al., 2017).

The NHEJ pathway is further divided into two pathways: classical and alternative NHEJ pathways. However, since NHEJ is error-prone, in many settings, the end product of this pathway usually contains added or missing DNA sequences which may result in a nonfunctional coding sequence (Hug et al., 2016).

NHEJ is the predominant DSB repair pathway and is responsible for most DSB repairs throughout the cell cycle (Arnoult et al., 2017). NHEJ is dependent on Ku to thread onto DNA termini and thus enhancing the affinity of NHEJ enzymatic components which contain a nuclease, a ligase, and two polymerases (Mateos-Gomez et al., 2017). Intriguingly, each of these enzymatic components is unique for its capability in working on a broad range of incompatible DNA ends coupled with flexibility in loading order, leading to several possible junctional consequences from one DSB. The DNA end configurations can be directly ligated. However, if these ends are incompatible, they may be processed until configurations that are ligatable are achieved that are usually stabilized by no more than 4 bp of terminal micro-homology. DNA ends processing causes the addition or loss of nucleotides, accounting for the fact that original DNA sequences can rarely be restored after NHEJ repair of DSBs. Collectively, NHEJ is a DSB repair pathway with various enzymes and can result in multiple repair outcomes (Pannunzio et al., 2018).

### Homology-Directed Repair

The second DSB repair pathway is HDR. This mechanism has high fidelity but low incidence. An exogenous repair template is utilized to direct cleavage of the DNA by the targeting nuclease. This can increase the probability of homologous recombination (HR) by about 1,000-fold. Notably, HDR can be used to accurately edit the genome in various techniques, including conditional gene knockout, gene knock-in, gene replacement, and point mutations (Arnoult et al., 2017). The HDR pathway uses homologous donor DNA sequences from sister chromatids or foreign DNA to create accurate insertions, base substitutions between DSB sites or two DSBs, and other modifications. This kind of precise modification is significant to genomic engineering in order to achieve the desired effect (Lin et al., 2014). Sequences of sister chromatids or homologous chromosomes form the basis of HDR. Sister chromatids are only available in the S and G2 phase; thus, HDR is limited to these phases of the cell cycle (Branzei and Foiani, 2008).

**Figure 2:**
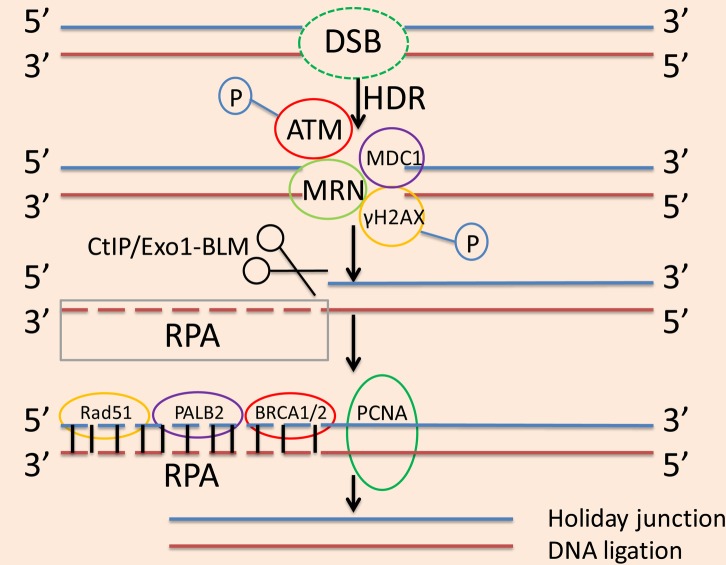
The process of the HDR pathway. In the HDR pathway, ATM phosphorylates H2AX, then MDC1 binds to this making γH2AX a site of accumulation at the area of DNA damage. The MRN complex localizes to the DSB, which exerts a stabilizing effect and inhibits chromosome breaks. After the initial stabilization of DSB, the 5′ exonuclease activity of CtIP or Exo1-BLM creates 3′SS overhangs, and RPA binds to these 3′SS overhangs. Rad51 works in conjunction with BRCA1 and BRCA2 as well as PALB2 to replace RPA and form filaments on the DNA. With the aid of PCNA, to synthesize the deleted DNA fragment. After the formation of the new DNA fragment, a Holliday junction is also formed, after which ligation is used to complete the reconstruction and restore the original DNA sequence.

Much research has been done on proteins involved in the HDR pathway. Ataxia telangiectasia mutated (ATM) phosphorylates H2A histone family member X (H2AX), then DNA damage checkpoint protein 1 (MDC1) binds to this making γH2AX a site of accumulation at the area of DNA damage (Marechal and Zou, 2013). The MRN complex is then localized to the DSB, which exerts a stabilizing effect and inhibits chromosome breaks. After the initial stabilization of the DSB, the 5′ exonuclease activity of C-terminal-binding protein-interacting protein (CtIP) or exonuclease 1-Bloom helicase (Exo1-BLM) creates 3′ single-stranded (3′SS) overhangs, and human replication protein A (RPA) binds to these 3′SS overhangs (Symington, 2014). Rad51 works in conjunction with breast cancer 1 and 2 proteins (BRCA1 and BRCA2) and BRCA2 molecular chaperones (Salsman et al., 2017) to replace RPA and forms filaments on the DNA. The reconstitution process is initiated by looking for repair templates or sister chromatids through the 3′ overhang of Rad51 (Buisson et al., 2014). With the aid of proliferating cell nuclear antigen (PCNA), to synthesize the deleted DNA fragment. After the formation of the new DNA fragment, a Holliday junction is also formed, after which ligation is completed and the original DNA sequence is restored, as shown in Figure 2 (Jasin and Rothstein, 2013).

The mechanism for repairing a DSB is not random; election of any repair mechanism will affect the results of genome editing. HDR is an uncommon form of DSB repair compared to NHEJ, but proper use of this repair mechanism for targeted genome editing can have a significant impact (Rothkamm et al., 2003). However, the availability of the HDR pathway is limited in undivided cells, which includes most cells *in vivo*. Therefore HDR-mediated genome editing methods are limited to *in vivo* applications (Nami et al., 2018).

## Methods for Enhancing Clustered Regularly Interspaced Short Palindromic Repeats/Cas9-Mediated Homologous-Directed Repair Efficiency

### Cell Cycle-Determining Pathway Components Determine Homologous-Directed Repair Selection and Efficiency

The key factor in selecting the repair pathway for the DSB is the phase of the cell cycle. Cells utilize the NHEJ method to repair DSBs occurring in G1, S, and G2 phases, while the HDR method is only available during the S and G2 phases (Sartori et al., 2007), with sister chromatids used as repair templates (Heyer et al., 2010; Gonzalez, 2016). Because these two repair strategies compete with each other, theoretically, suppressing NHEJ will improve the incidence of HDR (Arnoult et al., 2017). Based on this hypothesis, researchers blocked NHEJ either by chemical substances and small interfering RNA (siRNA) key proteins or knocked out key NHEJ effectors with siRNA and short hairpin RNA (shRNA) to inhibit the NHEJ pathway and increase the likelihood of the HDR pathway (Chu et al., 2015; Li et al., 2017, 2018a). In 2012, Srivastava and colleagues identified a small-molecule inhibitor of NHEJ, 5,6-bis-((E)-benzylideneamino)-2-mercaptopyrimidin-4-ol (SCR7) (Srivastava et al., 2012). Mechanistically, SCR7 blocks the NHEJ pathway by binding to DNA ligase IV, a key enzyme of the NHEJ pathway, in a concentration-dependent manner (Vartak and Raghavan, 2015). Specifically, it works by reducing the affinity of DNA ligase IV for DSBs (Gerlach et al., 2018). SCR7 binds to the DNA-binding domain of DNA ligase IV, thereby preventing DNA ligase IV from binding to the DNA ends, resulting in the elimination of the NHEJ pathway (Vartak and Raghavan, 2015). However, for other NHEJ proteins such as KU70/KU80, DNA-PKcs, and artemis, no suitable inhibitors have been found. Interestingly, experiments have shown that the addition of SCR7 does not improve HDR efficiency during genome editing. In contrast, coordinated expression of Cas9 in the HDR-dominant cell cycle is more efficient in inducing HDR than inhibition of NHEJ (Gerlach et al., 2018). Nonetheless, existing studies have shown that these manipulations may be difficult to perform, or the process of manipulation may cause greater damage to cells. This was evidenced by a study where the site cut by CRISPR-Cas9 was destroyed when using HDR to repair the DSB and could no longer be cut by CRISPR-Cas9 (Wang et al., 2015).

In turn, the cell cycle-determining pathway also affects the efficiency of HDR. A study found that Cas9-directed RNA ribonucleosides synergistically bind the cell cycle proteins to the pre-assembled Cas9 ribonucleoprotein (RNP) complex for direct nuclear transfection to improve the likelihood of HDR. In this approach, timed delivery of protein complexes can be controlled during the cell cycle phase of HDR (Jinek et al., 2013). This method can simultaneously transfect multiple Cas9 RNPs and donor DNAs with higher cell viability than DNA transfection (Kim et al., 2014; Zuris et al., 2015). These features enable powerful genome editing while reducing off-target effects. Importantly, this system maximizes the efficiency of HDR.

Nocodazole, an anti-tumor drug, acts to depolymerize microtubules, which are essential for cell mitosis; it can also interfere with the polymerization of microtubules and keep cells in the G2 or M phase of the cell cycle. Nocodazole treatment resulted in higher HDR selection when the Cas9 RNP dose was reduced (Lin et al., 2014). HDR is more prone to be selected with nocodazole treatment. One possibility to explain the higher efficiency is that Cas9 RNP targets multiple cells after synchronization with nocodazole. Another possibility is that the nuclear membrane is destroyed and Cas9 RNP can easily obtain DNA, leading to higher HDR efficiency. The high HDR efficiency upon treatment with nocodazole has no off-target editing and provides important advances in the development of scar-free genetic modification (Lin et al., 2014).

According to the current research, HDR of the Cas9 system has been used to knock-in genetic material. For example, *CXCR4* can be knocked in and knocked out by electroporation of Cas9 RNPs (Schumann et al., 2015). In addition, the work of Tu et al. has demonstrated that CRISPR/Cas9 nickase genome editing can efficiently result in a deletion of the RB1 gene in human embryonic stem cells (Tu et al., 2018). However, HDR of the Cas9 system does not guarantee successful genetic knock-in all the time. For example, although Cas9 RNP can mediate successful knock-in of specific nucleotides to *CXCR4* and *PD-1* in primary T cells, this is accompanied by a relatively higher incidence of off-target effects, rendering the efficiency of such genetic knock-in less significant in T cells compared with other cells (Schumann et al., 2015). Therefore, a detailed evaluation of the off-target effects of Cas9 RNP is needed. In addition, further investigation to resolve these off-target effects to make the Cas9 system more efficient is also deserved.

### Improving Homologous-Directed Repair Efficiency by Expressing Key Proteins of Homologous Recombination

Subsequent studies have found that further improvements in directing HDR selection may require regulation of related proteins or key factors in the HDR or NHEJ pathways (Humbert et al., 2012). These related proteins, referred to as key HDR factors, can switch DNA repair from NHEJ to HDR by stimulating these key HDR factors (Bozas et al., 2009). Moreover, it seems that HDR stimulation is a more effective way of precise knock-in than NHEJ inhibition.

#### Recombination Protein A (Rad) Family Members

When foreign DNA is integrated into the chromosome, members of the Rad family (Rad50, Rad51, Rad52, etc.) play an indispensable role (Shao et al., 2017). Rad52 is an important homologous recombinant protein, and its complex with Rad51 plays a key role in HDR, mainly involved in the regulation of foreign DNA in eukaryotes (Di Primio et al., 2005; Kalvala et al., 2010). Key steps in the process of HR include repair mediated by Rad51 and strand exchange. The current model assumes that the formation of Rad51 requires the interaction of Rad52 (Ma et al., 2018). In particular, researchers suggest that co-expression of Rad52 with CRISPR/Cas9 nucleases can significantly enhance the likelihood of HDR (Di Primio et al., 2005; Shao et al., 2017; Van Chu et al., 2018). As detected by genome editing assays, co-expression of these proteins increased the likelihood of HDR by approximately three-fold. Studies have shown that a Rad52-Cas9 fusion is a better choice for enhancing CRISPR/Cas9-mediated HDR and may be helpful for accurate genome editing studies. However, the Rad52-Cas9 fusion mediated by different donor templates showed different HDR enhancement efficiencies (Shao et al., 2017). In addition, RAD52 motif protein 1 (RDM1) is similar to RAD52; RDM1 can repair DSBs caused by DNA replication, prevent G2 or M cell cycle arrest, and improve HDR selection (Tong et al., 2018).

#### Fanconi Anemia Core Complex

Fanconi anemia (FA) is a recessive hereditary disease caused by a biallelic mutation in at least one of 22 genes (Palovcak et al., 2017). The FA core complex includes eight Fanconi anemia core complex (FANC) proteins that are members of the translesion synthesis polymerase family (Ceccaldi et al., 2016). Under many circumstances, DSBs can be repaired by HR proteins, including FANC proteins (Crossan et al., 2011; Stoepker et al., 2011). When there is no HR repair factor, a DSB can be joined by NHEJ repair (Palovcak et al., 2017).

FANC proteins are components of the FA core complex (Wang and Smogorzewska, 2015) with two biochemical activities: strand exchange (SE) and single-strand annealing (SSA). The published data suggest that the SE and SSA activities of FANC are closely associated and play a critical role in DSB repair, and cell-based DSB repair assays clearly demonstrate that FANC contributes to the DSB repairs (Benitez et al., 2018). In addition, the data also indicate that FANC plays a role in DSB repair by catalyzing SSA and/or SE (Leung et al., 2012; Benitez et al., 2018).

FANC itself has different affinities to DNA, with high affinity to single-stranded DNA (ssDNA) and relatively low affinity to double-stranded DNA (dsDNA) (Yuan et al., 2012). Through its high affinity to ssDNA, FANC takes two ssDNAs together to form a dsDNA. Once dsDNA is formed, the low affinity of FANC results in the release of dsDNA products from dsDNA, triggering subsequent catalytic processes (Benitez et al., 2018).

#### Tumor Suppressor *p53*

The tumor suppressor gene *p53* can cause the production of mutant protein, usually in the DNA-binding domain, and is one of the most common mutant genes in cancer. *p53* acts as a transcription factor to activate or inhibit the target gene (Haigis and Dove, 2003). It also performs downstream regulation processes such as apoptosis, DNA repair, and DNA recombination. *p53* plays a direct role in DNA repair, including HR regulation; it affects the extension of new DNA, thereby affecting HDR selection (Gottifredi and Wiesmuller, 2018). *In vivo*, p53 binds to the nuclear matrix and is a rate-limiting factor in repairing DNA structure (Wiesmuller et al., 1996). The tumor suppressor *p53* regulates DNA repair processes in almost all eukaryotes *via* transactivation-dependent and -independent pathways, but only the transactivation-independent function of *p53* is involved in HR regulation. Thus, *p53* can act as a “molecular node” located at the intersection of the upstream signal cascade and downstream DNA repair and recombination pathways (Sengupta and Harris, 2005).

Current research indicates that the wild-type (WT) p53 protein can link DSBs to form intact DNA (Tang et al., 1999), as well as also exerting a role in inhibiting NHEJ (Akyuz et al., 2002). A study found that p53 interacts with HR-related proteins, including Rad51; p53 controls HR through direct interaction with Rad51 (Linke et al., 2003; Sengupta et al., 2003). The interaction between HR proteins (such as RAD51 and RAD54) and HR-DNA intermediates indicates that p53 acts directly in HR in the early and late stages of recombination (Sengupta and Harris, 2005). This direct effect of p53 can maintain the stability of the genome. In 1996, Mummembrauer et al. found that the core domain of p53 has intrinsic 3′-5′ exonuclease activity (Mummenbrauer et al., 1996), and according to the damage of the DSB, p53 can play a role in correcting the mismatch of nucleic acids and exchanging incomplete homologous sequences (Sengupta and Harris, 2005).

#### C-Terminal-Binding Protein-Interacting Protein

In order to improve the efficiency of genome editing *via* elevated HDR selection, researchers around the world have developed many different strategies to date. Research on the development of genome editing technology involving the CRISPR-Cas9 system usually includes DSBs introduced by endonucleases; these are then repaired by HDR or the like (Rouet et al., 1994).

CtIP is a key protein in the early stages of HR. The minimal N-terminal fragment of CtIP is called the HDR enhancer, which is used to stimulate HDR (Gutschner et al., 2016). Increased rates of HDR can be achieved by fusing Cas9 to the N-terminal domain of CtIP, allowing CtIP to enter the cleavage site and increase transgene integration *via* HDR. HDR stimulation with Cas9 results in a two-fold or more increase in the frequency of targeted transgene integration, facilitating HDR-accurate genome editing (Charpentier et al., 2018).

### The Choice of Donor DNA Determines the Efficiency of Homologous-Directed Repair

Donor DNA is optional and can be either single-stranded or double-stranded. Studies have shown that the efficiency of HDR is determined by the donor DNA selected. If the donor DNA is double-stranded, after pairing with the invading genomic strand, it can begin to replicate by the action of the polymerase. If the donor DNA is single-stranded, the process of repairing the DSB is relatively easy (Song and Stieger, 2017). However, HDR is not highly efficient in all cells and is very inefficient in certain types of cells, such as induced pluripotent stem cells (iPSCs). To increase the HDR efficiency of these particular types of cells, cyclin D1 (CCND1) is involved in G1 to S conversion during the cell cycle, while nocodazole is a G2 to M phase synchronizer. The addition of these two components increased HDR efficiency by 30% in a study. In summary, the study found that the choice of DNA donor is closely related to HDR efficiency (Zhang et al., 2017).

Studies have also shown that if the donor DNA selected is single-stranded, the process of repairing a DSB will be relatively easy; however, the choice of the single-stranded donor will also have a significant impact on HDR efficiency. It has been proven that when Cas9-initiated HDR is used with a short single-stranded oligodeoxynucleotide pair, it can act on many genes. However, conditional null alleles are produced at the locus and are less efficient when applied on a large scale (Lanza et al., 2018). Conversely, long single-stranded oligodeoxynucleotides are matched for efficient high-throughput processes of large numbers of conditional alleles. Of course, no matter which single-stranded DNA is used as a donor, it is necessary to first screen for sequence errors in the HDR locus and randomly insert the donor sequence into the genome (Lanza et al., 2018). In addition, researchers also found that using overlapping single guide RNA (sgRNA) and single-stranded oligonucleotide-mediated HDR can improve HDR efficiency (Jang et al., 2018).

## Conclusions and Perspectives

In view of the CRISPR/Cas9-mediated genome editing strategy, the CRISPR/Cas9-NHEJ genome editing method is common, but CRISPR/Cas9-HDR is infrequent (Gerlach et al., 2018). The main reasons for this are the low efficiency of HDR and the poor availability of exogenous DNA as a repair template, which seriously affect HDR as an accurate method of genome editing (Li et al., 2018b). Current methods of enhancing HDR selection include using chemicals, inhibiting NHEJ, and regulating the cell cycle. However, these methods face many challenges (Ye et al., 2018).

HDR and NHEJ are different types of genome editing methods, but both are genome editing techniques for repairing DSBs. Previous research has shown that NHEJ is more error-prone when repairing DSBs, but in fact, recent studies have demonstrated that NHEJ repair is performed after Cas9 cuts the target position, and the process is repeated until an error occurs, which prevents Cas9-mediated DNA cleavage (Zaboikin et al., 2017). Therefore, errors that are prone to occur in NHEJ are not errors that occur at the outset. In fact, NHEJ is a key strategy for stabilizing the genome, which plays an important role in the repair of DSBs (Lu et al., 2018). Although HDR is more accurate, it has a specific cycle limit. When it is unavailable, NHEJ is still relied on to repair the DSB, but it is converse in the time period when the HDR method can be used, which implies it is the obvious advantage. Hence, in order to improve HDR selection, studies need to be done how to direct selection of HDR as the most effective way to repair the DSB in the future studies (Hu et al., 2018).

In addition to the commonly used CRISPR/Cas9-NHEJ and CRISPR/Cas9-HDR genome editing methods, there are also genome editing methods such as CRISPR/Cas12a-, CRISPR/Cas13-, and CasX-NHEJ/HDR. The CRISPR/Cas12a system may provide a means for inducing genomic alterations through HR, and complementation can convert repair from NHEJ to HR. Cas13 has been used to degrade mRNA and thus antagonize viral RNA replication (Schindele et al., 2018). In addition, CasX is a fundamentally distinct RNA-guided genome editing system that uses unique structures to generate staggered DSBs in DNA at sequences complementary to a 20-nucleotide segment of its guide RNA, making it a third enzyme family that is functionally distinct from Cas9, Cas12a, and Cas13 (Liu et al., 2019).

Of course, we still need a relatively neutral and critical attitude toward these technologies. Although they are widely used in many scenarios, other genome editing technologies have also been used in certain circumstances. For example, researchers have found that homology-mediated end joining (HMEJ)-based methods produce higher knock-in efficiency in HEK293T cells and primary astrocytes. Also, this method achieved transgenic integration in monkey and mouse embryos, which is more effective than NHEJ and HR methods (Yao et al., 2017). Therefore, in future genome editing applications, in addition to considering the Cas9-mediated HDR system for knocking in genetic material, HMEJ-based strategies can also be considered for a variety of applications to generate animal models and targeted gene therapy.

## Author Contributions

X-DT, W-TM, and D-KC designed the structure of this review. X-DT, FG, and M-JL wrote the first version of the manuscript. Q-LF helped revise the manuscript. All authors have reviewed the final version of the manuscript.

### Conflict of Interest Statement

The authors declare that the research was conducted in the absence of any commercial or financial relationships that could be construed as a potential conflict of interest.
